# Sustained Three-Year Remission Following Anti-IL-6 Therapy in a Patient With TAFRO (Thrombocytopenia, Anasarca, Fever, Reticulin Fibrosis, and Organomegaly) Syndrome After Initial Misdiagnosis: A Case Report

**DOI:** 10.7759/cureus.108233

**Published:** 2026-05-04

**Authors:** Javier E Moyano Navarro, David D Terrones Huamán, Julio J Barrios Aedo, Pablo F Moreno Mandarachi, Carlos Pachas

**Affiliations:** 1 Internal Medicine, Edgardo Rebagliati Martins National Hospital, Lima, PER; 2 Medicine, Universidad San Martin de Porres, Lima, PER; 3 Internal Medicine, School of Medicine, Universidad Científica del Sur, Lima, PER; 4 Pathology, Edgardo Rebagliati Martins National Hospital, Lima, PER

**Keywords:** anasarca, castleman disease, interleukin-6 inhibitor, sustained remission, systemic inflammatory disorder, tafro syndrome, thrombocytopenia, tocilizumab

## Abstract

TAFRO syndrome is an infrequent and severe systemic inflammatory condition marked by thrombocytopenia, anasarca, fever, reticulin fibrosis/renal dysfunction, and organomegaly. Although tocilizumab has shown clinical efficacy in inducing initial disease control, evidence on sustained long-term remission beyond three years following anti-interleukin-6 therapy remains limited. We present a 46-year-old female who was admitted with worsening edema, gingival bleeding, and severe thrombocytopenia, initially misdiagnosed as idiopathic thrombocytopenic purpura with no clinical response. A comprehensive diagnostic workup demonstrated multisite serosal effusions (pleural fluid accumulation, pericardial fluid accumulation, and abdominal free fluid), enlarged cervical lymph nodes, grade 1-2 reticulin myelofibrosis with megakaryocytic hyperplasia, Castleman disease-like features on nodal histopathological examination, and increased serum interleukin-6 levels (19.2 pg/mL). The patient fulfilled all major and multiple minor diagnostic criteria for TAFRO syndrome. Treatment consisted of combination therapy with cyclophosphamide, corticosteroids, plasmapheresis, and tocilizumab administered biweekly for a total of eight doses. Complete resolution of thrombocytopenia, anemia, and inflammatory markers was observed after five cycles of tocilizumab. Comprehensive clinical, laboratory, and radiological surveillance showed sustained complete remission for more than three years, without evidence of disease relapse. This report highlights the potential role of tocilizumab-based therapy in achieving prolonged clinical remission in selected patients with TAFRO syndrome, particularly when interpreted within the overall clinical and laboratory context.

## Introduction

TAFRO syndrome is a rare and severe systemic inflammatory disorder, with an estimated incidence of approximately 0.9-4.9 cases per million individuals in Japan. It is characterized by thrombocytopenia, anasarca, fever, reticulin fibrosis/renal dysfunction, and organomegaly [1-4]. The 2019 revised diagnostic criteria require all three major components (anasarca, thrombocytopenia, and systemic inflammation) and at least two minor criteria, including Castleman disease-like features on lymph node biopsy, reticulin myelofibrosis, mild organomegaly, and progressive renal insufficiency [1,3]. Castleman disease is a heterogeneous group of lymphoproliferative disorders characterized by lymph node enlargement and systemic inflammatory manifestations, particularly in its multicentric form (iMCD), which shares overlapping clinical and histopathological features with TAFRO syndrome [2,3]. In this context, the relationship between TAFRO syndrome and Castleman disease remains controversial. Some researchers consider it a distinct subtype of iMCD due to overlapping histopathological features, while others classify it as a separate clinical entity based on its markedly different clinical presentation and aggressive course [2,5].

The disease mechanism involves hypercytokinemia with disruption of inflammatory pathways, primarily interleukin-6 (IL-6) and vascular endothelial growth factor (VEGF), resulting in increased vascular permeability and systemic inflammation [2,3]. The overall prognosis remains poor, with approximately one-third of patients succumbing to the disease within 24 months of diagnosis and a two-year survival rate of only 66.5% [5]. Management remains challenging. Systemic corticosteroids are commonly used as first-line therapy; however, many patients require additional immunosuppressive agents such as tocilizumab or rituximab [3,6,7]. While anti-IL-6 therapies such as tocilizumab and siltuximab have demonstrated efficacy in achieving early disease control [7,8], evidence for sustained remission beyond three years, with thorough clinical, laboratory, and imaging follow-up, remains limited [7,9].

We present a case of TAFRO syndrome that achieved sustained complete remission for over three years following tocilizumab-based therapy. This case represents one of the longest documented follow-ups with comprehensive clinical, laboratory, and imaging monitoring and highlights the potential for durable disease control with early anti-IL-6 therapy in patients with elevated IL-6 levels.

## Case presentation

A 46-year-old female presented to her primary care physician with diffuse headache, progressive extremity edema, and gingival bleeding, ongoing for approximately two months. Her medical history was notable for hypothyroidism, managed with levothyroxine for the past four years. Family history was unremarkable. The referring physician initially suspected idiopathic thrombocytopenic purpura and administered three intravenous pulses of methylprednisolone. However, the patient’s gingival bleeding persisted, and the edema failed to improve. Consequently, she was admitted to our hospital for further evaluation and management.

Upon admission, the patient was alert and oriented to person, place, and time. Vital signs were as follows: blood pressure of 180/90 mmHg, heart rate of 95 beats per minute, respiratory rate of 18 breaths per minute, oxygen saturation of 98% on room air, and a body temperature of 37.6 °C. Physical examination revealed cervical lymphadenopathy, bilateral lower limb pitting edema (++/+++), diminished vesicular breath sounds in the lower thirds of both hemithoraces, and abdominal distension with shifting dullness. Laboratory results showed anemia, severe thrombocytopenia, leukocytosis, prolonged coagulation times, hypoalbuminemia, and elevated levels of alkaline phosphatase (ALP) and C-reactive protein (CRP) (Table 1). 

**Table 1:** Laboratory data on admission. Hb, hemoglobin; PLT, platelet count; WBC, white blood cell count; ANC, absolute neutrophil count; PT, prothrombin time; INR, international normalized ratio; APTT, activated partial thromboplastin time; TT, thrombin time; CRP, C-reactive protein; ALP, alkaline phosphatase; LDH, lactate dehydrogenase.

Parameter	Reference range	Value at admission
Hematology		
Hb (g/dL)	11.5–16	8.4
PLT (k/µL)	130–400	28
WBC (k/µL)	4–11	19.32
ANC (k/µL)	1.5–7.5	16.14
Blood coagulation		
PT (seconds)	10.5-13	16.6
INR	0.85-1.15	1.25
APTT (seconds)	25-37	57.4
TT (seconds)	16-21	15.7
Fibrinogen (mg/dL)	200–400	652
Blood chemistry		
CRP (mg/dL)	0–1	20.53
Creatinine (mg/dL)	0.5–0.8	0.99
Urea (mg/dL)	21.6–55.2	114.27
Albumin (g/dL)	3.2-4.8	2.75
Total protein (g/dL)	6.60-8.30	4.91
ALP (U/L)	46-116	592
LDH (U/L)	120-246	284

**Figure 1:**
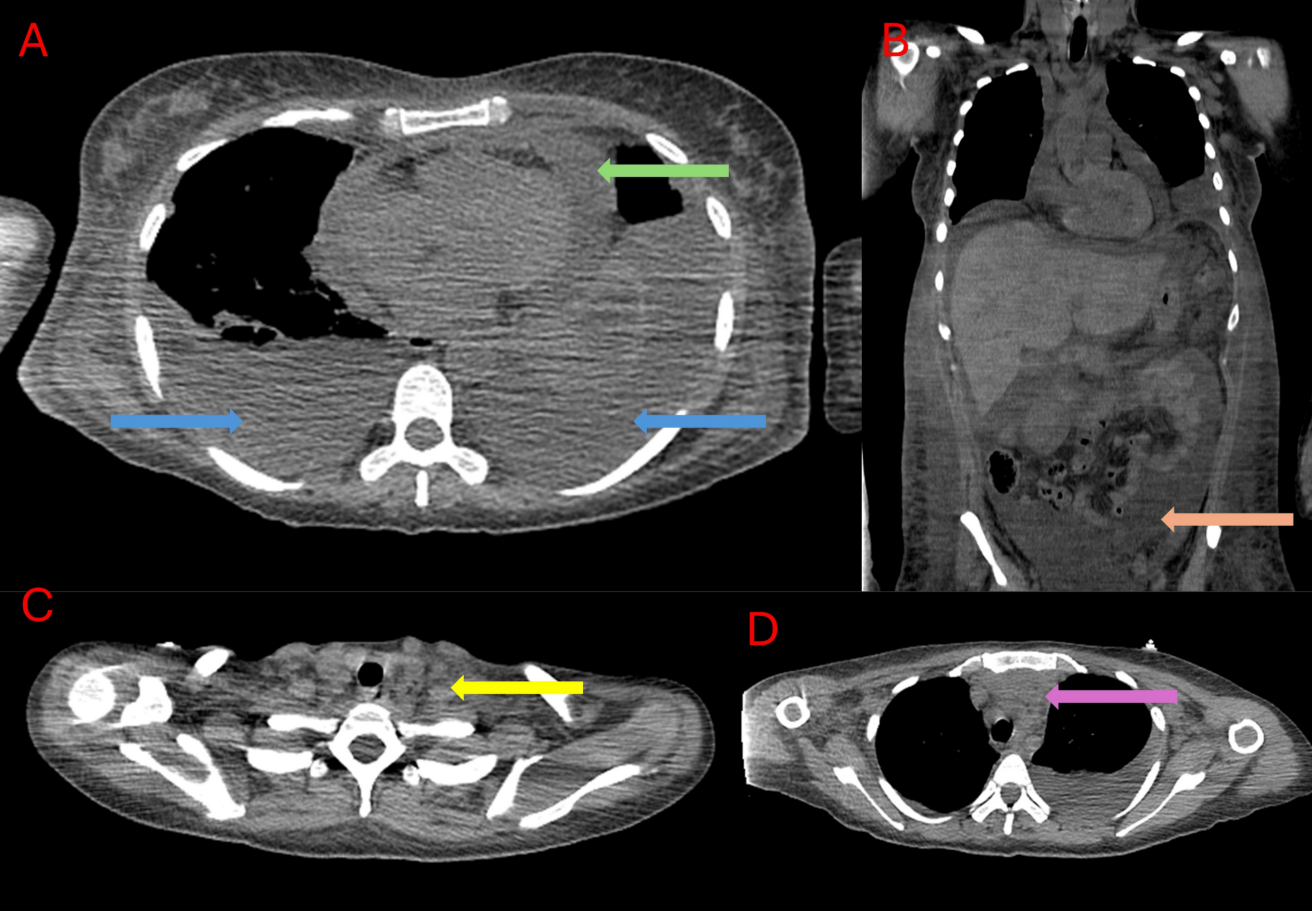
Computed tomography (CT) scan captured at admission. (A) Chest CT image showing bilateral pleural effusion (blue arrows) and pericardial effusion (green arrow). (B) Abdominal CT image showing ascites (orange arrow). (C) Cervical CT image showing multiple cervical lymphadenopathies (yellow arrow). (D) Chest CT image showing a poorly defined prevascular mediastinal mass (purple arrow).

A computed tomography (CT) scan performed after admission demonstrated bilateral cervical, axillary, and mediastinal lymphadenopathies; signs of polyserositis, including pericardial and bilateral pleural effusions and ascites; as well as a prevascular mediastinal mass suggestive of thymic hyperplasia (Figure 1). This pattern, combined with laboratory findings, raised suspicion for TAFRO syndrome rather than isolated ITP. Thoracocentesis and paracentesis were carried out for both diagnostic and therapeutic purposes, revealing negative cytology for malignancy and low adenosine deaminase (ADA) levels.

**Figure 2:**
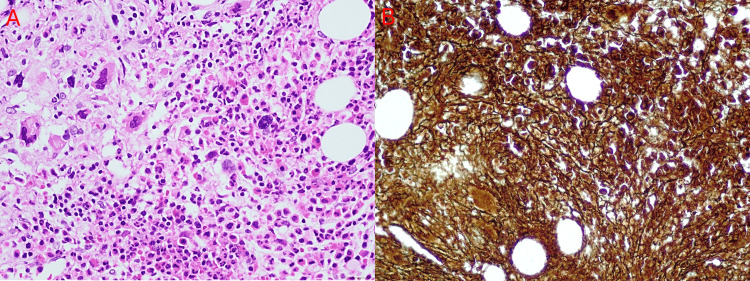
Bone marrow biopsy. (A) Hematoxylin and eosin-stained bone marrow section showing marked hypercellularity with increased megakaryocytes. (B) Reticulin staining of the bone marrow revealing grade 1–2 reticulin fibrosis.

On hospital day 6, serological and antigen tests for hepatitis viruses, human T-lymphotropic virus type 1 (HTLV-1), and the TORCH panel yielded negative results. Immunological evaluation showed normal complement levels (C3: 149 mg/dL; C4: 28 mg/dL), and a broad panel of autoantibodies returned negative results, including anti-centromere antibodies (ACA), anti-mitochondrial antibodies (AMA), anti-smooth muscle antibodies (ASMA), anti-cardiolipin antibodies, and the anti-treponemal test. Rheumatoid factor (RF) levels were within normal limits (8.20 IU/mL), while antinuclear antibodies (ANA) were detected at a low titer of 1:80, which is considered borderline and non-specific in the absence of clinical correlation. Notably, serum beta-2 microglobulin was elevated at 3.17 mg/L. By hospital day 20, a bone marrow biopsy was performed, which demonstrated grade 1-2 reticulin myelofibrosis associated with megakaryocytic hyperplasia (Figure 2). Flow cytometry of peripheral blood and bone marrow showed no pathological lymphocyte or plasma cell infiltration.

**Figure 3:**
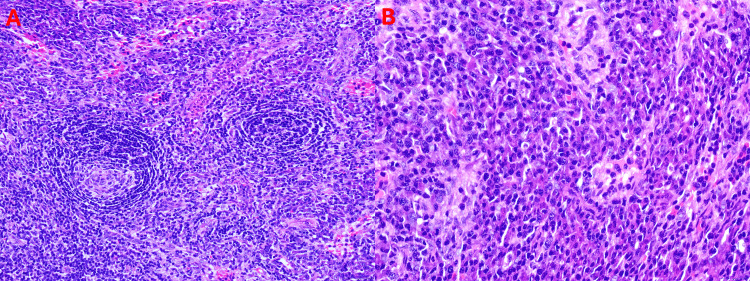
Cervical Lymph Node Biopsy (A) Low-power view showing partial architectural distortion, atrophic germinal centers, and marked interfollicular plasmacytosis. (B) High-power view demonstrating mild endothelial proliferation and sinusoidal dilatation.

By hospital day 27, histopathological examination of the cervical lymph node biopsy revealed findings consistent with Castleman disease-like features, possibly corresponding to the hypervascular pattern described in TAFRO-associated iMCD (Figure 3).

**Figure 4:**
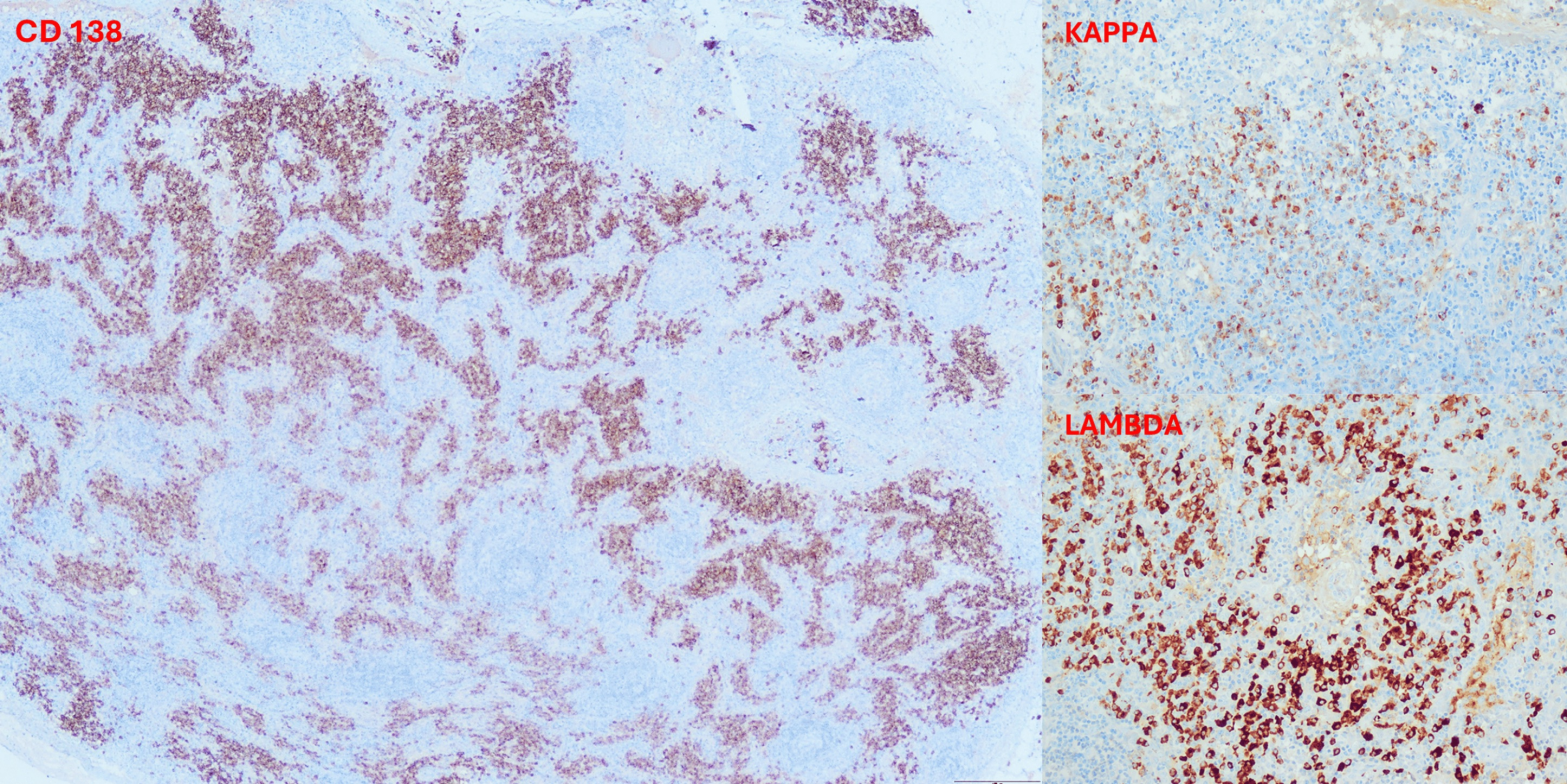
Immunohistochemical staining showing CD138-positive plasma cells with a polytypic kappa and lambda light chain expression (kappa-to-lambda ratio of 1:5).

Immunohistochemistry revealed CD138-positive plasma cells with a polytypic pattern (Figure 4), as well as the presence of CD3-positive T lymphocytes and CD20-positive B lymphocytes. Staining for human herpesvirus 8 (HHV-8) was negative. Concurrently, interleukin-6 levels were elevated at 19.2 pg/mL (normal <7 pg/mL), while HHV-8 and HIV serologies were negative. Serum immunoglobulin profiling showed IgG at 1,598 mg/dL, IgA at 358 mg/dL, and IgM at 58 mg/dL, along with a markedly elevated beta-2 microglobulin level of 5.06 mg/L, suggesting ongoing immune activation.

Based on the 2019 updated diagnostic criteria, the patient met all three major and three of the four minor criteria for TAFRO syndrome, including anasarca (pleural and pericardial effusions and ascites), thrombocytopenia, and systemic inflammation (body temperature of 37.6 °C and elevated C-reactive protein ≥2 mg/dL), along with reticulin myelofibrosis, mild organomegaly, and Castleman disease-like features on lymph node biopsy.

On hospital day 35, treatment with cyclophosphamide (750 mg/m² intravenously) was initiated. One week later, the patient received a single dose of intravenous immunoglobulin (IVIG, 0.4 g/kg) in combination with high-dose methylprednisolone (1 g/day for three consecutive days) and underwent plasmapheresis. Despite these interventions, the clinical response was limited, with persistent cytopenias and ongoing systemic symptoms. Due to elevated IL-6 levels, tocilizumab (8 mg/kg intravenously every two weeks) was initiated for a total of eight doses. The patient subsequently exhibited a marked clinical improvement, and by the fifth dose of tocilizumab, leukocytosis, anemia, and thrombocytopenia had resolved (Table 2). Thalidomide (100 mg orally once daily) was initiated concurrently for the management of grade 1-2 reticulin myelofibrosis, with subsequent stabilization of hematologic parameters and no evidence of progression of marrow fibrosis during follow-up.

**Table 2:** Evolution of laboratory parameters over the course of tocilizumab therapy. Hb, hemoglobin; PLT, platelet count; WBC, white blood cell count; ANC, absolute neutrophil count; CRP, C-reactive protein; ALP, alkaline phosphatase; LDH, lactate dehydrogenase; AST, aspartate aminotransferase; ALT, alanine aminotransferase; GGT, gamma-glutamyl transferase; BU, unconjugated bilirubin; BC, conjugated bilirubin; PT, prothrombin time; INR, international normalized ratio; APTT, activated partial thromboplastin time; TT, thrombin time; NA, not available.

		During tocilizumab treatment					Post-treatment		
Values	Normal range	Post-first dose	Post-second dose	Post-third dose	Post-fourth dose	Post-fifth dose	1-year follow-up	2-year follow-up	3-year follow-up
Hematology									
Hb (g/dL)	11.5–16	8.6	9.8	12.2	13.4	13.5	13.4	12.6	12.9
PLT (k/µL)	130–400	13	54	128	235	215	244	270	253
WBC (k/µL)	4–11	2.29	10.71	5.98	5.07	4.25	5.14	5.65	5.38
ANC (k/µL)	1.5–7.5	1.02	7.76	4.36	2.27	1.6	2.25	2.84	3.01
Blood chemistry									
CRP (mg/dL)	0–1	1.33	0.4	0.4	0.4	0.4	<0.4	NA	NA
Creatinine (mg/dL)	0.5–0.8	0.47	0.42	0.45	0.55	0.57	0.63	0.66	0.74
Urea (mg/dL)	21.6–55.2	28.44	40.66	42.28	39.78	44.08	34.71	43.38	NA
Albumin (g/dL)	3.2-4.8	3.3	3.35	4.13	4.09	4.20	4.44	4.59	4.44
Total protein (g/dL)	6.60-8.30	6.46	5.71	6.68	6.18	6.10	6.75	6.99	7.08
Globulin (g/dL)	2-3.5	3.16	2.36	2.55	2.09	1.9	2.31	2.4	NA
ALP (U/L)	46-116	311	206	110	65	162	113	NA	NA
LDH (U/L)	120-246	480	283	240	158	164	140	146	138.24
AST (U/L)	10-34	32	19	38	30	32	28	18	17.55
ALT (U/L)	10-49	NA	56	189	138	67	51	33	15.10
GGT (U/L)	5-38	204	150	100	52	230	43	NA	NA
Total bilirubin (mg/dL)	0.3-1.2	1.48	0.77	0.92	0.56	0.82	0.49	0.42	0.49
BU (mg/dL)	0.2-1.0	0.77	0.38	0.51	0.34	0.59	0.36	0.31	0.43
BC (mg/dL)	0.1-0.3	0.71	0.39	0.41	0.22	0.23	0.13	0.11	0.06
Blood coagulation									
PT (seconds)	10.5-13	NA	NA	12.0	10.70	NA	10.70	NA	NA
INR	0.85-1.15	NA	NA	1.06	0.94	NA	0.94	NA	NA
APTT (seconds)	25-37	NA	NA	29.80	25.50	NA	30.30	NA	NA
TT (seconds)	16-21	NA	NA	19.90	16.90	NA	14.80	NA	NA
Fibrinogen (mg/dL)	200–400	NA	NA	131.4	249.4	NA	356	NA	NA

**Figure 5:**
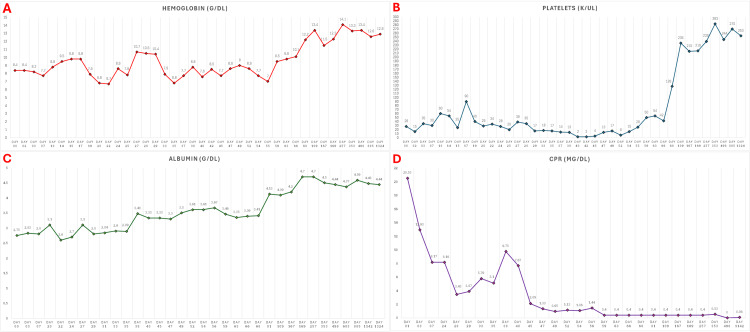
Longitudinal follow-up of laboratory markers during and after tocilizumab-based treatment. (A) Hemoglobin levels showed progressive recovery, reaching sustained normalization during follow-up. (B) Platelet counts remained critically low during the first weeks of treatment but increased markedly after the fifth dose of tocilizumab, with a sharp rise by day 68 and sustained recovery thereafter. (C) Albumin levels improved gradually, reflecting recovery from hypoalbuminemia. (D) CRP levels decreased rapidly after treatment initiation and remained persistently suppressed throughout follow-up.

Clinical and laboratory follow-up demonstrated a sustained response at one month, one year, two years, and over three years post-treatment, with stable platelet counts, hemoglobin, and albumin levels, as well as normalization of CRP levels (Figure 5).

**Figure 6:**
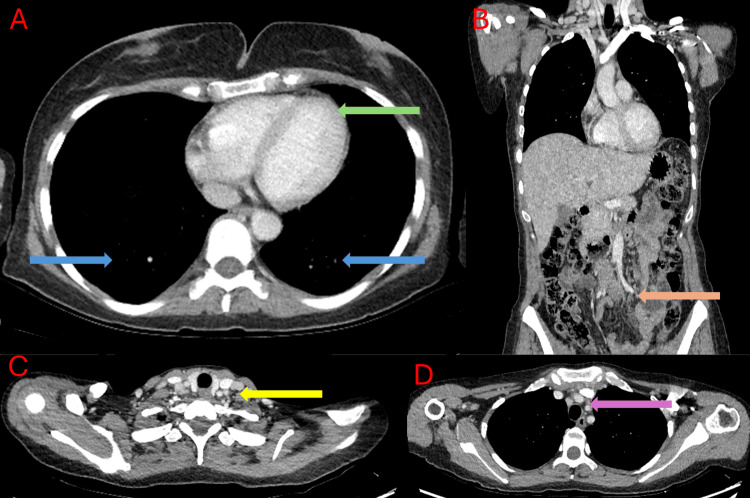
One-year follow-up contrast-enhanced CT showing complete radiological remission. (A) Axial chest CECT image showing resolution of pleural effusion (blue arrows) and pericardial effusion (green arrow). (B) Coronal abdominal CECT image demonstrating absence of ascites (orange arrow). (C) No evidence of cervical lymphadenopathy (yellow arrow). (D) Complete regression of the previously noted prevascular mediastinal mass (red arrow).

Serial follow-up imaging with contrast-enhanced CT (CECT) demonstrated complete resolution of ascites and regression of the prevascular mediastinal mass. Additionally, no new pathological findings were identified, and cervical lymphadenopathy was no longer observed (Figure 6). The patient remained in complete remission throughout the follow-up period as of November 2025, with no signs of disease recurrence, without the need for maintenance immunosuppressive therapy due to sustained clinical improvement.

## Discussion

This case report presents a 46-year-old female with TAFRO syndrome who achieved complete disease remission after tocilizumab treatment, showing persistent clinical and laboratory improvement over three years of follow-up. Our patient met all three major criteria and three minor criteria according to the 2019 updated definition. Although the recorded temperature (37.6 °C) does not meet conventional fever thresholds (≥38 °C), it meets the definition of systemic inflammation under TAFRO criteria (fever >37.5 °C or elevated C-reactive protein ≥2 mg/dL) [1,3], supporting the diagnosis. 

The clinical presentation of our patient is consistent with the typical manifestations of TAFRO syndrome described in the literature. However, unlike the patient reported by Moy et al., who developed disseminated intravascular coagulation, renal dysfunction, and neurologic complications leading to death despite treatment [10], our patient maintained a stable coagulation profile and avoided catastrophic outcomes. This contrast may reflect earlier diagnosis and prompt initiation of IL-6 blockade, underscoring the critical importance of early intervention in TAFRO syndrome. The characteristic laboratory findings in our patient, such as elevated alkaline phosphatase (592 U/L), hypoalbuminemia (2.75 g/dL), and normal immunoglobulin levels, aligned with the typical profile of TAFRO syndrome rather than with iMCD-NOS, which typically presents with polyclonal hypergammaglobulinemia [2,3,5].

The diagnostic challenge of TAFRO syndrome is well documented in the literature. Ma et al. described a case initially misdiagnosed as liver cirrhosis due to persistent abdominal distension, highlighting the difficulty of early diagnosis [11]. Similarly, Qiao et al. reported a patient initially diagnosed with mixed connective tissue disease, whose delayed diagnosis led to clinical deterioration [12]. In our case, the initial suspected diagnosis was idiopathic thrombocytopenic purpura, and the patient received methylprednisolone pulse therapy with no clinical improvement. The diagnostic workup involved a comprehensive evaluation, including imaging studies, bone marrow biopsy, lymph node biopsy, and systematic exclusion of differential diagnoses. Malignancy was ruled out through negative lymph node cytology, absence of monoclonal proteins, and a polytypic plasma cell pattern on immunohistochemistry. In addition, lymph node histopathology revealed Castleman disease-like features with vascular changes, including endothelial proliferation and sinusoidal dilatation, which are consistent with patterns described in TAFRO-associated iMCD, where hypervascular or mixed histological subtypes have been reported [3,5].

Autoimmune etiologies were excluded based on a borderline antinuclear antibody (ANA) titer (1:80, considered non-specific), normal complement levels, and absence of disease-specific autoantibodies. Infectious causes were also excluded, with negative serologies for HIV and HHV-8. The presence of a prevascular mediastinal mass, suggestive of thymic hyperplasia, is consistent with findings reported by Morita et al., who described a fatal TAFRO case with an anterior mediastinal mass that, on autopsy, revealed fibrous thymic tissue. Notably, anterior mediastinal masses have been detected in up to 64% of TAFRO patients on CT imaging [13]. These observations highlight a potential association between TAFRO syndrome and mediastinal involvement, warranting further investigation to clarify the underlying pathophysiology. In our case, complete resolution of the mediastinal mass at one-year follow-up (Figure 6) suggests a reactive process, likely thymic hyperplasia, rather than a neoplastic lesion. This finding carries important clinical implications, as mediastinal masses in TAFRO syndrome may mimic lymphoma or thymoma on imaging, potentially leading to unnecessary invasive procedures or misdiagnosis.

Management strategies for TAFRO syndrome vary widely in the literature due to the rarity of the disease and the lack of randomized controlled trials. Our patient received combination therapy consisting of cyclophosphamide, methylprednisolone, plasmapheresis, intravenous immunoglobulin, and tocilizumab. The decision to initiate tocilizumab was guided by elevated IL-6 levels (19.2 pg/mL) and supporting evidence on the efficacy of anti-IL-6 therapy in TAFRO syndrome [6-8]. In a retrospective review, Fujimoto et al. reported that while rituximab extended the interval before additional treatment compared to tocilizumab, there were no significant differences in overall survival between groups, suggesting that salvage therapies were effective in many patients who failed second-line treatments [7]. In our case, tocilizumab was highly effective, leading to the resolution of thrombocytopenia, anemia, anasarca, and inflammatory markers by the fifth dose. Masaki et al. emphasized that TAFRO syndrome requires urgent medical intervention due to its aggressive nature and potential for rapid deterioration [2,4]. The intensive management approach described by Masui et al., including circulatory, fluid, and respiratory support, further highlights the importance of coordinated multidisciplinary care in severe cases [14]. Although our patient presented with significant manifestations such as polyserositis and severe thrombocytopenia, she did not experience life-threatening complications such as hemorrhage or multiorgan failure, which have been reported in other cases [10,13,14].

The administration of multiple concurrent therapeutic modalities, including cyclophosphamide, corticosteroids, plasmapheresis, and tocilizumab, makes it difficult to isolate the specific contribution of each intervention to the observed clinical remission. However, the temporal association between the initiation of tocilizumab and the subsequent sustained resolution of hematologic and inflammatory parameters, alongside markedly elevated baseline IL-6 levels, suggests a relevant role for IL-6 blockade in this case. While further prospective studies are needed to define the optimal combination or sequence of therapies in TAFRO syndrome, this case highlights the potential role of anti-IL-6 therapy in selected patients, interpreted within the overall clinical and laboratory context.

## Conclusions

We report a case of TAFRO syndrome that achieved sustained complete remission over three years following tocilizumab-based therapy, representing one of the longest documented follow-up periods with comprehensive clinical, laboratory, and imaging surveillance. Despite an initially severe presentation, complete resolution of hematologic and inflammatory abnormalities was achieved without recurrence. This case supports the potential effectiveness of anti-IL-6 therapy in achieving durable responses when initiated promptly in selected patients, interpreted within the broader clinical and laboratory context. Given the limited long-term data beyond three years and the aggressive nature of TAFRO syndrome, this report underscores the importance of early recognition, thorough diagnostic assessment, and timely, individualized therapeutic decision-making, while highlighting the need for further prospective studies.
